# Supplementary material for: Circulating Chemerin Levels, but not the RARRES2 Polymorphisms, Predict the Long-Term Outcome of Angiographically Confirmed Coronary Artery Disease
Source: Int J Mol Sci. 2019 Mar 7;20(5):1174. doi: 10.3390/ijms20051174 (PMC6429458; doi:10.3390/ijms20051174)
Supplement: Supplementary file 1 [file ijms-20-01174-s001.pdf]

Supplementary Table 1. Genome-wide significance for the association between *RARRES2* gene polymorphisms and chemerin levels

SNP	position	Gene	Function	Population	MA	MAF	HWE	R^2 (%)	β	SE	*P* value [a]	Conditional *P* value [b]
rs57888387	150307155	*ACTR3C*	intron	TWB	A/G	0.275	0.683	0.027	0.028	0.003	9.37×10^{-17}	0.274
rs7781827	150311769	*ACTR3C*	intron	TWB	C/T	0.404	0.897	0.018	0.021	0.003	1.20×10^{-11}	0.802
rs17837498	150312590	*ACTR3C*	intron	TWB	A/G	0.398	0.988	0.018	0.021	0.003	9.72×10^{-12}	0.872
rs7806429	150316304	*RARRES2*	3' UTR	TWB	C/T	0.386	1.000	0.022	0.023	0.003	3.48×10^{-14}	0.271
				CAD	C/T	0.379	0.986	--	0.020	0.011	0.082	--
rs1962004	150323292	*RARRES2*	3' UTR	TWB	A/G	0.268	0.608	0.034	0.032	0.003	1.54×10^{-20}	0.403
				CAD	A/G	0.257	0.454	0.006	0.024	0.012	0.044	--
rs3735167	150342466	*RARRES2*	5'UTR (-781)[c]	TWB	T/C	0.268	0.649	0.035	0.033	0.003	2.35×10^{-21}	Lead SNP
				CAD	T/C	0.254	0.290	0.007	0.024	0.012	0.038	--
rs28432021	150349146	*LOC107986858*	intron	TWB	C/G	0.264	0.552	0.031	0.031	0.004	3.50×10^{-14}	0.859
rs1132261	150362898	*LOC105375563*	NC transcript	TWB	G/A	0.410	0.929	0.019	0.021	0.003	3.47×10^{-12}	0.350

TWB: Taiwan Biobank population; CAD: coronary artery disease; UTR: untranslated region; SNP: single nucleotide polymorphism; NC: noncoding, MA: minor allele; MAF: minor allele frequency; HWE: Hardy–Weinberg equilibrium; SE: standard error.

[a] *P* value: Adjusted for sex, age, body mass index, and current smoking status; Conditional *P* value: further adjustment of the lead SNP rs3735167.

[b] analyzed only in TWB;

[c] With transcription factor binding sites

Supplementary Table 2. *RARRES2* gene polymorphisms and chemerin levels in a cardiovascular health examination population previously reported [7]

SNPs	position	Gene	Location	Function	MA	MAF	HWE	β (SE)	R^2 (%)	*P* value[a]
rs7806429	150316304	*RARRES2*	3' (+22014)	3'UTR	C/T	0.410	0.841	0.027 (0.008)	0.017	0.001
rs1962004	150323292	*RARRES2*	3' (+15021)	3'UTR	A/G	0.283	0.864	0.035 (0.008)	0.025	3.80×10^{-5}
rs4721	150338437	*RARRES2*	3' (+3248)	Intron	T/G	0.476	0.973	0.023 (0.008)	0.013	0.002
rs17173608	150339575	*RARRES2*	3' (+2110)	Intron	G/T	0.067	0.899	0.002 (0.015)	--	0.912
rs3735167	150342466	*RARRES2*	-781	5'UTR	T/C	0.282	1.000	0.038 (0.008)	0.026	5.27×10^{-6}
rs10244748	150344521	*RARRES2*	-2836	5'UTR	C/T	0.214	0.907	-0.013 (0.009)	--	0.174
rs10282458	150348213	*RARRES2*	-6528	5'UTR	A/G	0.283	0.896	0.038 (0.008)	0.024	$1.08 \times 1 0^{-5}$

Abbreviations as in Table 2.

[a] *P* values: adjusted for sex, age, body mass index, and current smoking status

Supplementary Table 3. Chemerin levels: stepwise linear regression analysis, including genotypes, in a cardiovascular health examination population previously reported [7]

Variables	β (SE)	R^2 (%)	*P* value
Body mass index (BMI)	0.010 (0.002)	0.064	< 0.0001
RARRES2 rs3735167 genotypes	0.038 (0.008)	0.026	< 0.0001
Age	0.002 (0.001)	0.026	< 0.0001
Current smoker	0.041 (0.014)	0.007	0.005
Sex	0.026 (0.012)	0.008	0.026

Abbreviations as in Table 2.

[a] The multiple linear regressions were adjusted for age, sex, current smoking status, BMI, and *RARRES2* rs3735167, rs7806429, and rs1962004 genotypes

Supplementary Table 4. Chemerin and C-reactive protein (CRP) levels according to the cardiovascular risk factors and severity of coronary artery disease (CAD)

		Chemerin			CRP		
		Circulating levels Means ± SD (N)	*P1* value [a]	*P2* value	Circulating levels Means ± SD (N)	*P1* value	*P2* value
Sex*	Male	130.56 ± 54.75 (387)	< 0.0001	< 0.0001	7.27 ± 20.17 (387)	0.830	0.472
	Female	159.06 ± 66.10 (93)			8.25 ± 20.10 (92)		
Current smoker#	No	134.28 ± 57.99 (363)	0.136	0.010	6.70 ± 19.17 (362)	0.026	0.007
	Yes	141.67 ± 58.57 (117)			9.81 ± 22.87 (117)		
Hypertension	No	121.80 ± 51.92 (105)	0.002	0.007	7.68 ± 18.23 (105)	0.841	0.916
	Yes	140.07 ± 59.23 (375)			7.40 ± 20.69 (374)		
Diabetes mellitus	No	121.73 ± 44.79 (267)	< 0.0001	< 0.0001	6.09 ± 15.71 (267)	0.024	0.018
	Yes	154.07 ± 67.37 (213)			9.19 ± 24.58 (212)		
Obesity	No	132.86 ± 59.53 (207)	0.118	0.042	9.79 ± 26.64 (207)	0.744	0.995
	Yes	138.52 ± 57.08 (273)			5.69 ± 13.03 (272)		
Dyslipidemia	No	137.16 ± 61.46 (179)	0.717	0.764	7.67 ± 17.30 (178)	0.066	0.092
	Yes	135.11 ± 56.08 (292)			6.40 ± 19.54 (292)		
CAD severity	SVD	121.68 ± 41.49 (134)			5.28 ± 11.15 (134)		
	DVD	141.49 ± 64.04 (135)	0.015	0.014	9.52 ± 26.52 (135)	0.794	0.725
	TVD	141.77 ± 61.83 (211)			7.53 ± 19.84 (210)		

[a] *P1*: Not adjusted; *P2:* Multiple linear regressions adjusted for age and sex.

Supplementary Table 5. Combined chemerin and CRP levels associated with various clinical and biochemical parameters in CAD patients

Subgroup (number)		Total (481)	Low + Low (347)	Low + High or High + Low (103)	High + High (31)	*P* Value [a]
Anthropology	Age (years)	65.6 ± 11.3	65.1 ± 11.1	66.1 ± 11.1	70.2 ± 13.2	0.046
	Body mass index (kg/m^2)	26.0 ± 4.0	25.8 ± 3.6	26.9 ± 5.0	24.8 ± 4.0	0.270
	Hypertension (%)	78.2	76.2	80.6	90.3	0.158
	Diabetes mellitus (%)	44.3	37.7	62.1	58.1	0.0002
	Current smokers (%)	24.3	23.8	26.2	25.8	0.145
	Dyslipidemia (%)	60.9	62.0	64.1	35.5	0.153
Renal function	Creatinine (mg/dL)	1.1 (0.9 - 1.3)	1.0 (0.9 - 1.1)	1.4 (1.0 - 2.3)	1.4 (1.2 - 1.8)	< 0.0001
	eGFR (mL/min/1.86 m^2)	69.7 ± 24.5	76.8 ± 18.3	50.1 ± 28.5	50.9 ± 26.5	< 0.0001
Blood cell counts	Leukocyte counts (10^3/μL)	6.6 ± 2.1	6.3 ± 1.6	6.9 ± 2.0	9.7 ± 4.4	< 0.0001
	Hematocrit (%)	40.8 ± 5.4	42.1 ± 3.9	38.2 ± 6.7	34.9 ± 7.3	< 0.0001
	Platelet counts (10^3/μL)	211.9 ± 60.9	207.9 ± 48.6	218.7 ± 75.2	230.8 ± 109.1	0.007
Inflammatory marker	CRP (mg/L)	2.5 (1.2 - 4.3)	2.0 (1.0 - 3.2)	3.2 (1.9 - 7.1)	26.8 (16.2 - 84.2)	< 0.0001
	Chemerin (ng/mL)	124.4 (94.4 - 162.8)	106.8 (89.1 - 132.7)	187.8 (169.2 - 225.7)	211.3 (179.6 - 261.5)	< 0.0001

Abbreviations as in Table 1.

[a] *P* values were adjusted for age, sex, body mass index, and current smoking status.

Supplementary Table 6. Association between *RARRES2* genotypes and chemerin levels in previous genome-wide association studies (GWASs) and in our studies

References	Study Population	Ethnicity/Country	No.	Characteristics	GWAS	SNPs in array	Lead *RARRES2* SNP	R^2 (%)	*P* value
14	San Antonio Family Heart Study	Mexican-American	858	Randomly sampled without regard to phenotype or disease status	+	542,944	NA	NA	NA
13	Sorbs cohort, KORA, PPP-Botnia study, LIFE Leipzig study	Germany	2791	Non-diabetes	+	2,023,499	rs7806429	0.020	7.79×10^{-14}
15		Austria	495	Underwent angiography [a]	+	Cardio-metabolic chips (130,909)	rs3735167	NA	0.002
This study	Taiwan Biobank	Han Chinese	2197	Relatively healthy general population	+	614,820	rs3735167	0.035	2.35×10^{-21}
7		Han Chinese	612	Cardiovascular health examination	-	--	rs3735167	0.026	5.27×10^{-6}
This study		Han Chinese	481	Angiographically confirmed CAD	-	--	rs3735167	0.007	0.038

SNP: single nucleotide polymorphism; NA: not available.

[a] Including patients with significant coronary artery disease (CAD) (247) and nonsignificant CAD (248).

Supplementary Figure 1A and 1B

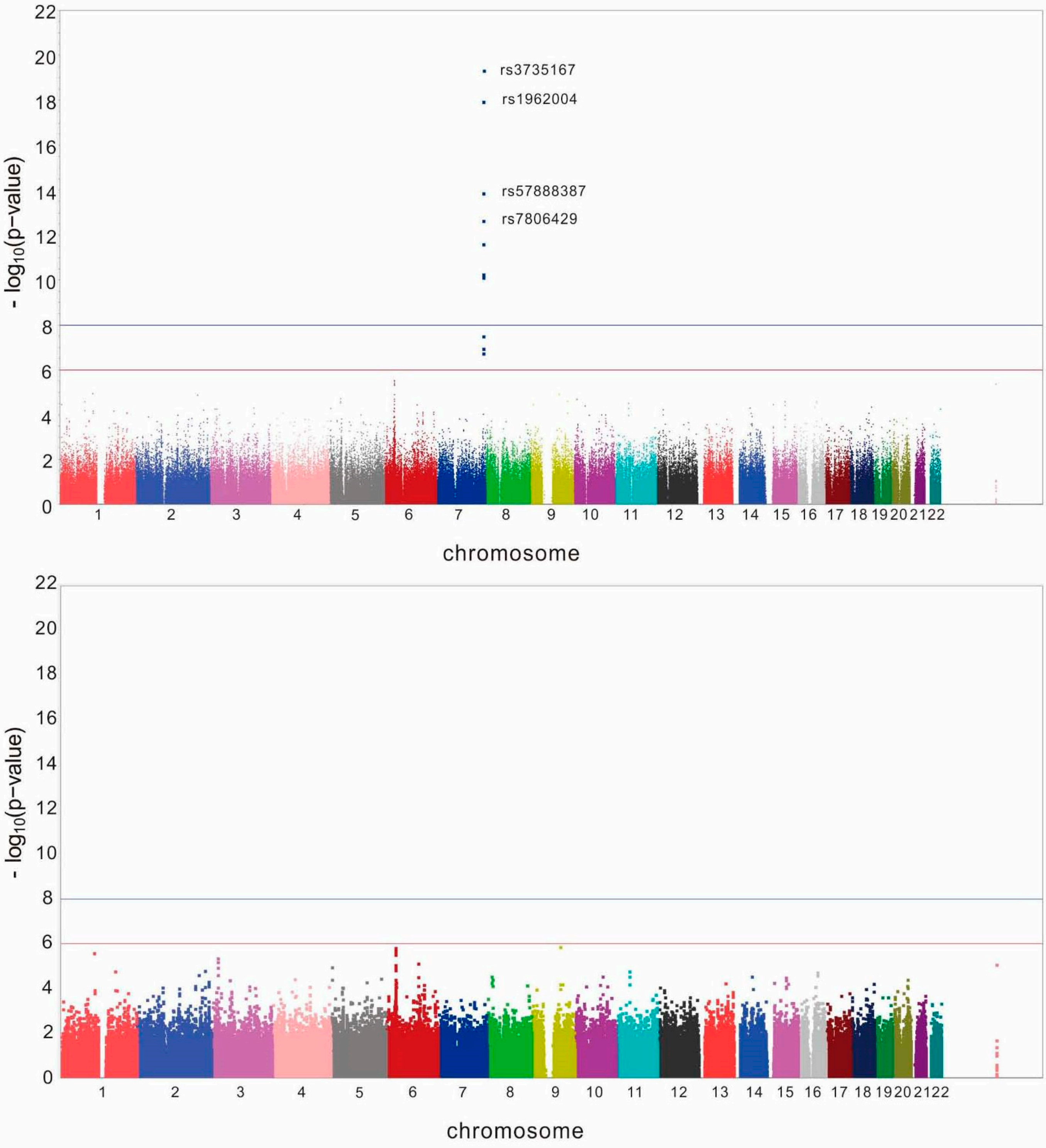

Supplementary Figure 2

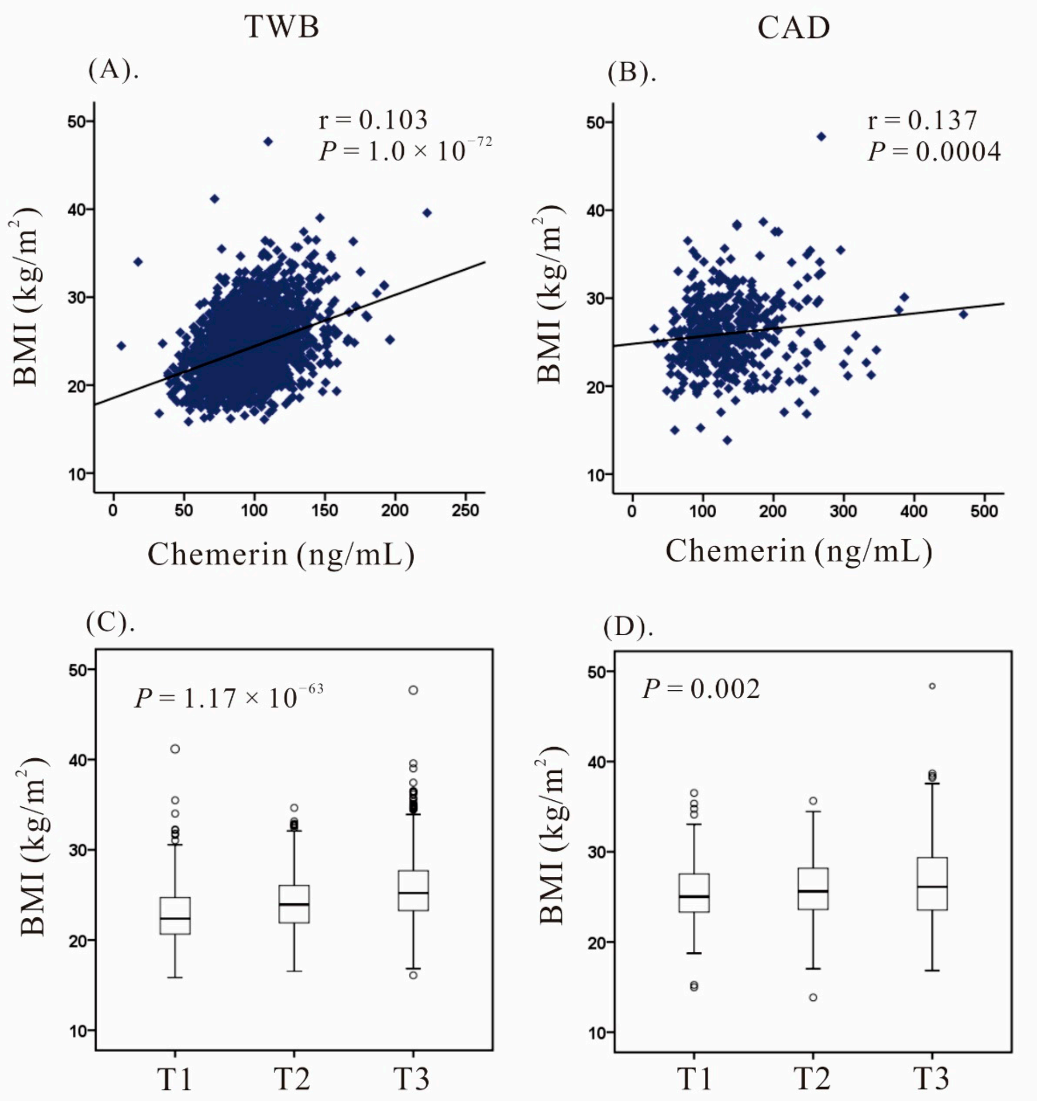

T1 = low < Chemerin ≦ 85.90
T2 = 85.90 < Chemerin ≦ 104.92
T3 = 104.92 < Chemerin ≦ max

T1 = low < Chemerin ≦ 102.47
T2 = 102.47 < Chemerin ≦ 147.16
T3 = 147.16 < Chemerin ≦ max

Supplementary Figure 3

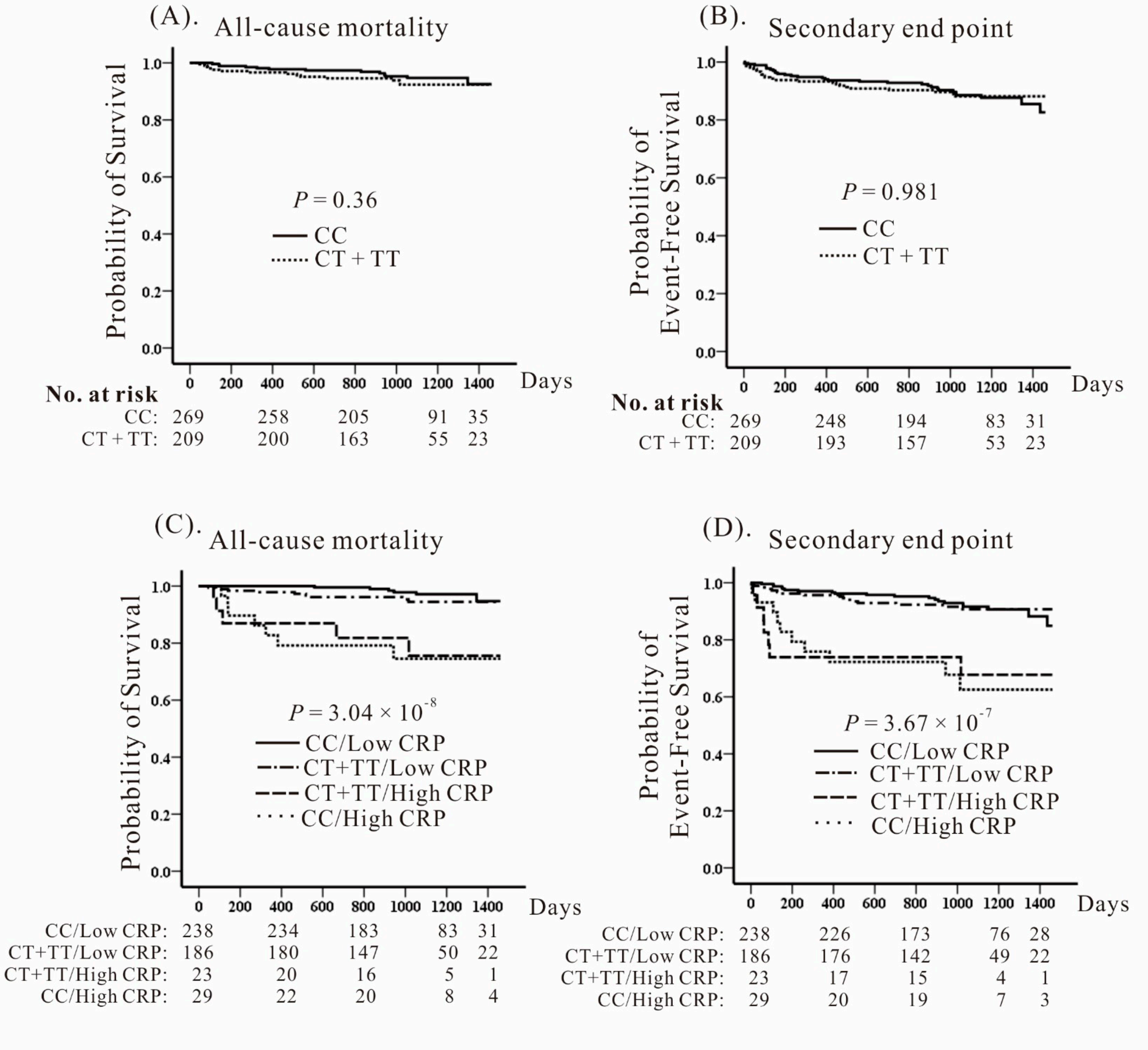

Supplementary Figure Legend

Supplementary Figure 1. Manhattan plots of the genome-wide association study for chemerin levels. Manhattan plots of the genome-wide association study for chemerin levels without (A) or with (B) conditional analysis of the rs3735167 polymorphism. The general linear model was used to examine the association between single nucleotide polymorphisms and chemerin levels after adjustment for age, sex, body mass index, and current smoking status. The blue and red dashed horizontal lines revealed a $-\log_{10}$ (*P* value) of 8 and 6, respectively.

Supplementary Figure 2. Association between BMI and circulating chemerin levels in TWB population and patients with angiographically confirmed coronary artery disease (A, B); Association between BMI and tertiles of circulating chemerin levels were presented as Box and whisker plots analysis (C, D).
P value: Adjusted for sex, age and current smoking status. Abbreviations as in Table 1.

Supplementary Figure 3. Kaplan–Meier curves of the cumulative incidence of primary and secondary endpoints. Kaplan–Meier curves of the cumulative incidence of primary and secondary endpoints according to the rs3735167 genotypes. No significant difference was found between the rs3735167 genotype and long-term outcomes of CAD patients in the total study population (A, B) or in subgroups according to C-reactive protein (CRP) levels (C, D).
